# Evolution of the Plant Reproduction Master Regulators LFY and the MADS Transcription Factors: The Role of Protein Structure in the Evolutionary Development of the Flower

**DOI:** 10.3389/fpls.2015.01193

**Published:** 2016-01-06

**Authors:** Catarina S. Silva, Sriharsha Puranik, Adam Round, Martha Brennich, Agnès Jourdain, François Parcy, Veronique Hugouvieux, Chloe Zubieta

**Affiliations:** ^1^CNRS, Laboratoire de Physiologie Cellulaire & Végétale, UMR 5168Grenoble, France; ^2^Laboratoire de Physiologie Cellulaire & Végétale, University of Grenoble AlpesGrenoble, France; ^3^Commissariat à l´Energie Atomique et aux Energies Alternatives, Direction des Sciences du Vivant, Laboratoire de Physiologie Cellulaire & Végétale, Institut de Recherches en Technologies et Sciences pour le VivantGrenoble, France; ^4^Laboratoire de Physiologie Cellulaire & Végétale, Institut National de la Recherche AgronomiqueGrenoble, France; ^5^European Synchrotron Radiation Facility, Structural Biology GroupGrenoble, France; ^6^European Molecular Biology Laboratory, Grenoble OutstationGrenoble, France; ^7^Unit for Virus Host-Cell Interactions, University of Grenoble Alpes-EMBL-CNRSGrenoble, France; ^8^Faculty of Natural Sciences, Keele UniversityKeele, UK

**Keywords:** evolution, SEPALLATA3, AGAMOUS, LEAFY, protein crystallography, small angle X-ray scattering, homology modeling

## Abstract

Understanding the evolutionary leap from non-flowering (gymnosperms) to flowering (angiosperms) plants and the origin and vast diversification of the floral form has been one of the focuses of plant evolutionary developmental biology. The evolving diversity and increasing complexity of organisms is often due to relatively small changes in genes that direct development. These “developmental control genes” and the transcription factors (TFs) they encode, are at the origin of most morphological changes. TFs such as LEAFY (LFY) and the MADS-domain TFs act as central regulators in key developmental processes of plant reproduction including the floral transition in angiosperms and the specification of the male and female organs in both gymnosperms and angiosperms. In addition to advances in genome wide profiling and forward and reverse genetic screening, structural techniques are becoming important tools in unraveling TF function by providing atomic and molecular level information that was lacking in purely genetic approaches. Here, we summarize previous structural work and present additional biophysical and biochemical studies of the key master regulators of plant reproduction – LEAFY and the MADS-domain TFs SEPALLATA3 and AGAMOUS. We discuss the impact of structural biology on our understanding of the complex evolutionary process leading to the development of the bisexual flower.

## Introduction

**FIGURE 1:**
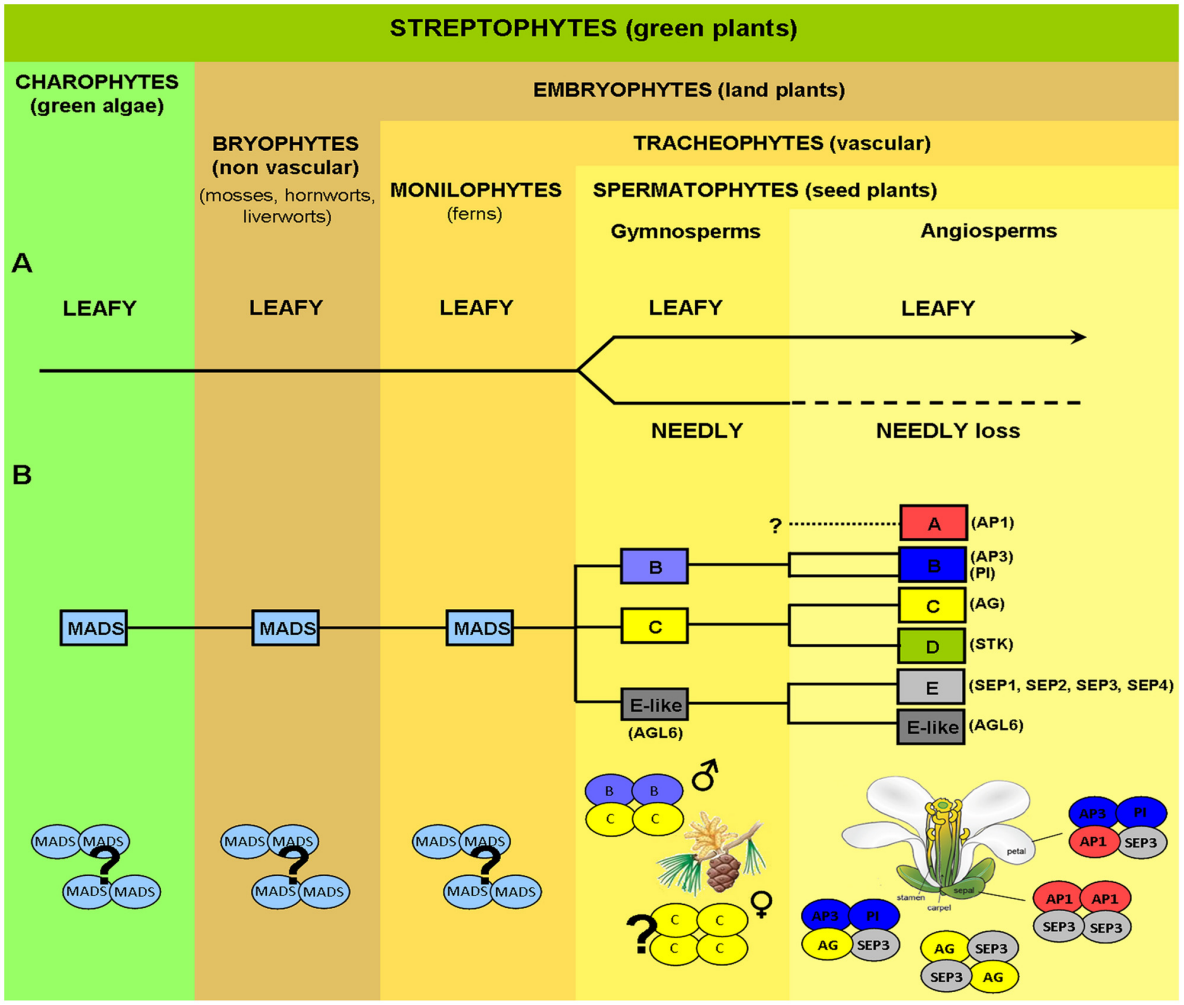
**Evolution of key genes controlling plant reproductive development.** **(A)** Evolution of *LEAFY* (*LFY*) from green algae to angiosperms. *LFY* exists mostly as a single-copy gene in all streptophytes (green plants), with the exception of gymnosperms where a *LFY*-like paralog, *NEEDLY* (*NLY*), originated after a major duplication event (the only possible exception being the genus *Gnetum*). In gymnosperms, *LFY* and *NLY* are consistently expressed in both male (pollen-bearing) and female (seed-bearing) cones, in a spatiotemporal coordinated manner. In the angiosperm lineage, *NLY* was subsequently lost, with *LFY* now regulating the expression of genes responsible for both the male and female organs in the unified bisexual flower. **(B)** MADS-box homeotic gene family. MADS-box genes are present in the most simple green algae and, as plants became more complex, the MADS-box gene family expanded via multiple duplication and specification events. Putative orthologs of class B, C, and E-like (*AGL6*) floral homeotic genes have been isolated from different gymnosperms (conifers, gnetophytes, ginkgophytes, and cycads) as shown schematically by yellow and blue colored ovals. In contrast, *SEP*-like genes, the second subfamily conferring E-class function, as well as A-class genes, seem to be absent in extant gymnosperms but are present in all angiosperms. In gymnosperms, expression patterns of putative B and C-class gene orthologs resemble those of B and C-class genes in angiosperms, with B-class genes being expressed on male reproductive organs, whereas C-class genes are expressed in both male and female organs. In gymnosperms C-class proteins alone or C and B-class proteins together seem capable of forming tetrameric complexes (without any additional partners), which define, respectively, the female and male organs in these organisms as indicated. In angiosperms tetramer formation is dependent on the SEPALATTA (E-class) TFs which act as hubs by mediating interactions among proteins from different floral homeotic classes, strictly determining floral organ identity. Question marks indicate uncertainty as to physiological oligomerisation state, AP1, APETALA1; AP3, APETALA3; PI, PISTILLATA; AG, AGAMOUS; STK, SEEDSTICK; SEP, SEPALLATA; AGL6, AGAMOUS LIKE 6.

The evolution of streptophytes (green plants), chronicled by the fossil record, follows a trajectory from simple green algae, to the earliest land plants (mosses, hornworts, liverworts), to free-sporing vascular plants (lycopsids including extant clubmosses, quillworts and spike mosses and monilophytes such as ferns and horsetails) and finally culminating with more complex seed plants (**Figure 1**). As the climate changed and became less favorable to spore-forming lycophtyes and monilophytes, spermatophytes (seed plants) were able to supplant these spore-forming vascular plants to become the majority of land plant species. The radiation of seed plants was due in large part to their ability to reproduce without the necessity of water for the dispersal of pollen or successful fertilization, as in the case of mosses and ferns. The reproductive adaptations in seed plants acted as a driver for terrestrial colonization and played a key role in their radiation across a wide range of habitats.

Extant seed plants are further divided into two sister groups, the gymnosperms and the angiosperms. Gymnosperms have naked seeds unprotected by a carpel and generally develop as the result of a single fertilization event. Exceptions exist as is the case of the genus *Ephedra* and *Gnetum* (Friedman, 1990; Friedman and Carmichael, 1996). In contrast, angiosperm seeds are enclosed and protected by the carpel and result from a double fertilization event that ensures the simultaneous development of the zygote and nutritive tissues, the endosperm (Lord and Russell, 2002). In addition to these variations in fertilization and seed development, the most striking difference between gymnosperms and angiosperms is the evolutionary innovation of the angiosperm flower. This novel arrangement joins the male and female organs into one reproductively competent structure. While the evolution of green plants from algae to seed plants follows a relatively smooth path in the fossil record, the evolution of the flower in angiosperms represents an evolutionary leap lacking an extensive step-wise fossil record. Since the time of Charles Darwin, the “abominable mystery” of flower origins and the unprecedented explosive radiation of angiosperm species have been the subject of extensive study and speculation (Burkhardt et al., 1985; Friedman, 2009).

In contrast to gymnosperm cones, which are unisexual and lack an enveloping perianth (sterile outer organs), angiosperm flowers have both male and female reproductive organs on a single axis surrounded by sepals and petals. A typical angiosperm flower is composed of four organs arranged in four concentric whorls. The outermost whorl contains the green protective sepals, followed by a whorl of petals involved in flower opening and pollinator attraction, the next whorl contains the stamens that produce pollen and constitute the male gametophyte, and finally the inner most whorl comprising the pistil, composed of one or more carpels, that contain the ovules. This basic floral architecture can vary across angiosperms. For example, basal angiosperms may contain tepals, sterile outer organs that cannot be differentiated into distinct sepals and petals. In addition, the number of flower parts and their arrangement around the central axis of the flower may vary as in orchids where the male and female organs are fused. However, the essential characteristic of the flower, co-localized male and female organs, is retained across all angiosperm species and acts as a defining trait.

### Angiosperm and Gymnosperm Evolution

One of the central questions in plant evolutionary developmental biology is how the flower, a bisexual compacted reproductive structure, evolved and what were the underlying molecular mechanisms for this dramatic morphological change. Extant gymnosperms and angiosperms separated ∼300 Mya (Zhang et al., 2004), with angiosperms quickly achieving an unprecedented level of species dominance, with over 350,000 extant species, in a dramatically short evolutionary timescale. However, simple morphological comparisons between gymnosperm cones and angiosperm flowers offer limited insight into flower evolution (Bateman et al., 2006; Frohlich and Chase, 2007). An understanding of the abrupt appearance of the flower from gymnosperm cones requires not only a fossil record to probe the changing morphologies of plant reproductive structures, but also a molecular basis derived from genome sequencing, molecular biology and structural biology. Impressive progress has been made in understanding the gene networks that regulate plant reproduction in angiosperms and, albeit to a lesser extent, also in gymnosperms. Due to extensive forward and reverse genetic studies (Coen and Meyerowitz, 1991; Saedler et al., 2001; Theissen and Saedler, 2001; Krizek and Fletcher, 2005) and whole genome sequencing in model plants such as thale cress (*Arabidopsis thaliana*), snapdragon (*Antirrhinum majus*) and petunia (*Petunia x hybrida*), as well as the large scale gene sequencing initiatives such as the 1000 plant genomes project (https://sites.google.com/a/ualberta.ca/onekp/home) and the complete sequencing and annotation of the first gymnosperm genome from Norway spruce (Nystedt et al., 2013), many of the genes which regulate the transition from vegetative to reproductive growth in angiosperms and gymnosperms have been identified.

### Gene Regulatory Networks Controlling Plant Reproductive Development

Despite the morphological difference between angiosperm and gymnosperm reproductive structures, a comparison of the genes responsible for male and female organ development demonstrates a high degree of conservation. Based on studies in angiosperm model plants such as *Arabidopsis*, development is switched from a vegetative to a reproductive program based on exogenous environmental and endogenous developmental signals such as plant age. This switch is orchestrated by the high level regulator of reproductive development, *LEAFY* (*LFY*), a gene that is conserved in gymnosperms and angiosperms (Vazquez-Lobo et al., 2007; Moyroud et al., 2010) and which has recently been identified in green algae, suggesting ancestral functions predating land plants (Sayou et al., 2014). Interestingly, while existing primarily as a single copy gene in most angiosperms, gymnosperms have two paralogous *LFY*-like genes- *LFY* and *NEEDLY* (*NLY*; Frohlich and Meyerowitz, 1997; Vazquez-Lobo et al., 2007), the only known exception being the gymnosperm genus *Gnetum* where *NLY* is absent (Frohlich and Meyerowitz, 1997; Frohlich and Parker, 2000). In addition to conservation of *LFY*, the genes that determine the identity of male and female reproductive organs, the MADS-box genes, are also present in both angiosperms and gymnosperms (Gramzow et al., 2010, 2014; Melzer et al., 2010; Wang et al., 2010). However, in contrast to gene loss in angiosperms as observed for *NLY*, the MADS-box genes have undergone multiple duplication events, leading to a more extensive gene network in angiosperms versus their sister gymnosperms (**Figure 1**). *LFY*, *NLY* and the MADS-box genes all encode transcription factors (TFs). These TFs act as master regulators and are able to direct extensive downstream gene networks. Recent work examining the function of LFY, NLY and the MADS TFs at the protein level has greatly advanced our understanding of how relatively small changes in a few key regulatory TFs can result in large differences at the morphological level of the organism. Current hypotheses point to changes in a few key genes, and the TFs they encode, as determining factors in the evolution of plant reproduction and the formation of the flower (Theissen, 2000, 2005; Zahn et al., 2005a,b; Theissen and Melzer, 2007; Melzer et al., 2010).

### The Role of LFY and LFY/NLY in Angiosperms and Gymnosperms

In angiosperms such as *Arabidopsis* or *Antirrhinum*, the switch to reproductive growth involves the conversion of the shoot apical meristem (SAM) to an inflorescence meristem (IM). The IM will in turn generate the floral meristem (FM) on its flanks. The development of a FM can be divided into two main steps (1) the formation of a specific zone within the IM, called the anlage, from which the FM will arise and (2) the growth of the FM primordia and subsequent differentiation into the floral organs. It is a balance between inflorescence identity genes such as *TERMINAL FLOWER 1* (*TFL1*) and FM identity genes such as *LFY* that determines the acquisition of flower identity. *TFL1* is predominantly expressed in the IM and acts as a repressor, preventing *LFY* and the MADS-box gene, *APETALA1* (*AP1*), expression (Liljegren et al., 1999). Increasing levels of LFY act as a committing step in FM identity, with LFY repressing expression of *TFL1* and inducing the expression of FM identity genes such as *AP1* (Parcy et al., 1998; Wagner et al., 1999; Kaufmann et al., 2010; Moyroud et al., 2010; Winter et al., 2011).

In gymnosperms, *LFY* and *NLY* expression patterns overlap in male and female cones early in development with expression patterns diverging later into mutually exclusive but complementary domains, resulting in higher *LFY* expression levels in male cones and higher *NLY* expression in female cones (Shindo et al., 2001; Dornelas and Rodriguez, 2005; Vazquez-Lobo et al., 2007). Originally, the *NLY* gene was thought to exclusively specify gymnosperm female reproductive structures (seed-bearing cone) in *Pinus radiata* (Mouradov et al., 1998), whereas its paralogous gene *LFY* appeared restricted to the male pollen-carrying cones (Mellerowicz et al., 1998). However, subsequent findings of *LFY* orthologs being expressed in female cones of gnetophytes and congeneric conifers (Carlsbecker et al., 2004; Dornelas and Rodriguez, 2005), demonstrated concurrent expression of both genes in male and female reproductive structures. Thus, *LFY* and *NLY* from gymnosperms are both necessary to act as regulators of male and female cone development, likely fulfilling a similar critical role in plant reproduction as the single copy angiosperm *LFY*.

### The Roles of the MADS-Box Genes and MADS TFs in Organ Identity

Once the FM is specified, LFY activates additional floral organ identity genes including the MADS-box genes *AP3*, *AG*, and *SEP3* (Weigel and Meyerowitz, 1993; Busch et al., 1999; Wagner et al., 1999; Lamb et al., 2002; Lohmann and Weigel, 2002; Winter et al., 2011). To date there is no direct evidence that gymnosperm LFY or NLY directly regulate MADS-box genes in gymnosperms as LFY does in angiosperms, although this is possible and warrants study. Once expressed, the overlapping patterns of the MADS-box genes will specify floral organ identity as outlined in the ABC(D)E model (Schwarz-Sommer et al., 1990; Coen and Meyerowitz, 1991) and for review see (Sablowski, 2010). In essence, the MADS-box genes can be divided into classes A-E with A+E genes necessary for sepal development, A+B+E genes specifying petals, B+C+E genes specifying stamen, C+E genes specifying carpels and D+E specifying ovules (Theissen and Saedler, 1995; Theissen, 2000; Honma and Goto, 2001; Ng and Yanofsky, 2001; Theissen and Saedler, 2001; Favaro et al., 2003). In *Arabidopsis* the class A genes are *APETALA1* (*AP1*) and *APETALA2* (*AP2*), class B genes are *APETALA3* (*AP3*) and *PISTILLATA* (*PI*), class C is *AGAMOUS* (*AG*) and class E are *SEPALLATA1,2,3,4 (SEP1,2,3,4)*. Except for *AP2*, all the floral homeotic genes in the ABC(D)E model encode MADS-domain TFs. The molecular mechanism of action of these proteins is explained by the floral quartet model, in which the A-E class genes encode TFs which are able to homo and heterotetramerise in specific combinations, resulting in the activation or repression of distinct downstream target genes and thus specifying floral organ identity (Honma and Goto, 2001; Theissen, 2001).

Gymnosperms possess B- and C-like MADS-box genes with their expression patterns resembling B- and C- class genes in angiosperms (Tandre et al., 1995; Sundstrom et al., 1999; Becker et al., 2002, 2003; Jager et al., 2003; Melzer et al., 2010; Wang et al., 2010; Gramzow et al., 2014). Indeed, several studies have described the expression of C-like genes in both male and female cones, while B-like gene expression appeared to be restricted to male cones (Sundstrom and Engstrom, 2002; Wang et al., 2010). Complementation studies have demonstrated that B and C homologs are well-conserved between gymnosperms and angiosperms as B and C genes from gymnosperms can nearly fully restore a wild type flower phenotype (Winter et al., 2002; Zhang et al., 2004). In addition, the gymnosperm MADS-domain TFs from the B and C class appear competent to form homo and heterotetramers, similarly to their angiosperm orthologs (**Figure 1**; Wang et al., 2010). Interestingly, the *SEP* subfamily members are absent in gymnosperms but are present in all major lineages of extant angiosperms (Zahn et al., 2005a). Based on phylogenetic analysis, the closest relative of the *SEP* subfamily is the *AGL6* subfamily, which is found in both angiosperms and gymnosperms (Becker and Theissen, 2003; De Bodt et al., 2003; Martinez-Castilla and Alvarez-Buylla, 2003; Nam et al., 2003; Zahn et al., 2005a). Similarly to class E *SEP* genes in angiosperms, *AGL6*-like genes are predominantly expressed in reproductive tissues in gymnosperms (for review see, Melzer et al., 2010) and represent the closest homologs to the *SEP* subfamily. Changes in the regulation of B and C class genes during evolution coupled with the appearance of the *SEP*-like genes and the dependence on the SEP TFs to form tetrameric MADS protein complexes, have been proposed to be crucial for the appearance of the bisexual flower. By requiring the SEP TFs to form transcriptionally active complexes with other homeotic MADS TFs, male and female organ identity may have become more easily co-regulated due to the multiple roles of the SEPs in specifying all reproductive organs.

The gene regulatory networks directing plant reproduction in gymnosperms and angiosperms are becoming more well-defined and the changes in key genes in gymnosperms and angiosperms which may be at the nexus of flower origins have been identified based on genetics studies in angiosperms and large scale sequencing initiatives in most plant lineages. However, only recently has the structure-function relationship of the proteins encoded by these key genes been determined. Here, we summarize available structural studies and provide new data to show how changes at the protein level in the key regulators LFY, NLY, and MADS-domain TFs potentially result in new functionality. Using biophysical data as a foundation, we probe the molecular mechanisms underlying the emergence and evolution of the novel reproductive architecture of the angiosperm flower and discuss how biochemistry and structural biology can provide new insights into evolutionary developmental biology.

## Materials and Methods

### Sequence Alignments

Sequence alignments were performed using the server NPS@ (Network Protein Sequence Analysis; Combet et al., 2000). Sequences were aligned with ClustalW (Thompson et al., 1994) using the default parameters for both pairwise alignment and multiple alignment sections. Where appropriate, secondary structure predictions were carried out with PREDATOR (DSSP) using the NPS@ server. Protein sequences used were obtained from GenBank and the 1000 Plants (1KP) initiative (http://www.onekp.com). Resulting alignments and secondary structure predictions were rendered with ESPript (Robert and Gouet, 2014).

**FIGURE 2:**
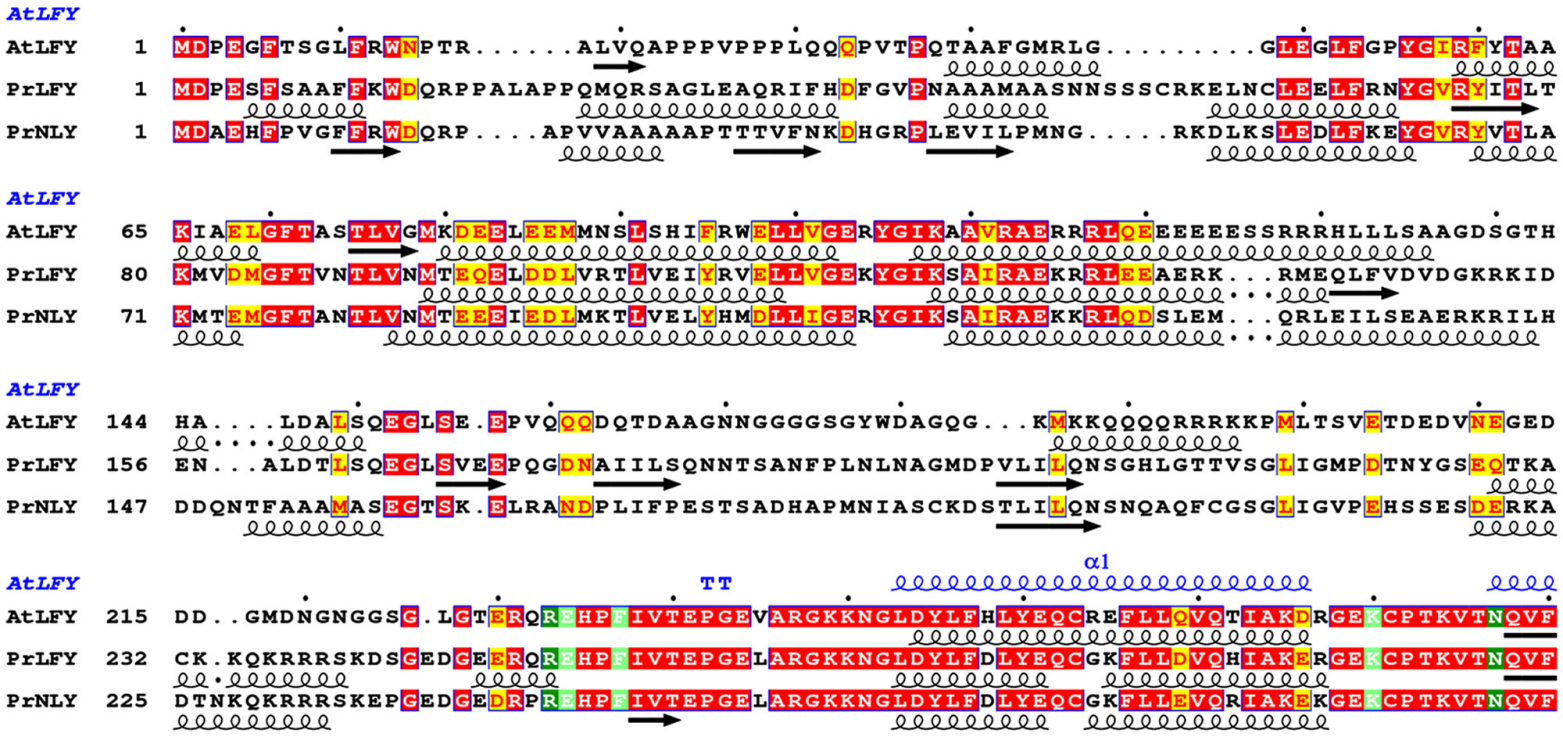
**Sequence alignment and secondary structure prediction of AtLFY, PrLFY, and PrNLY N-terminal domains, linker and start of conserved C-terminal DBD.** Aligned amino acid sequences of AtLFY (*Arabidopsis thaliana* LFY), PrLFY (*Pinus radiata* LFY, PRFLL) and PrNLY (*Pinus radiata* NLY), with respective secondary structure prediction indicated in black below each respective sequence; α-helices are represented by spirals and β-strands by arrows, all other regions are predicted to be unstructured. Sequence numbering is shown on the left and dots mark every 10th residue for the first sequence. Highly conserved regions are boxed, with similar residues represented in red against a yellow background, invariant residues represented against a red background and non-conserved residues indicated in black. Partial AtLFY DBD secondary structure, as derived from its X-ray structure (PDB 2VY1), is shown in blue above the sequences. Alignment was prepared with NPS@ (Combet et al., 2000).

**FIGURE 3:**
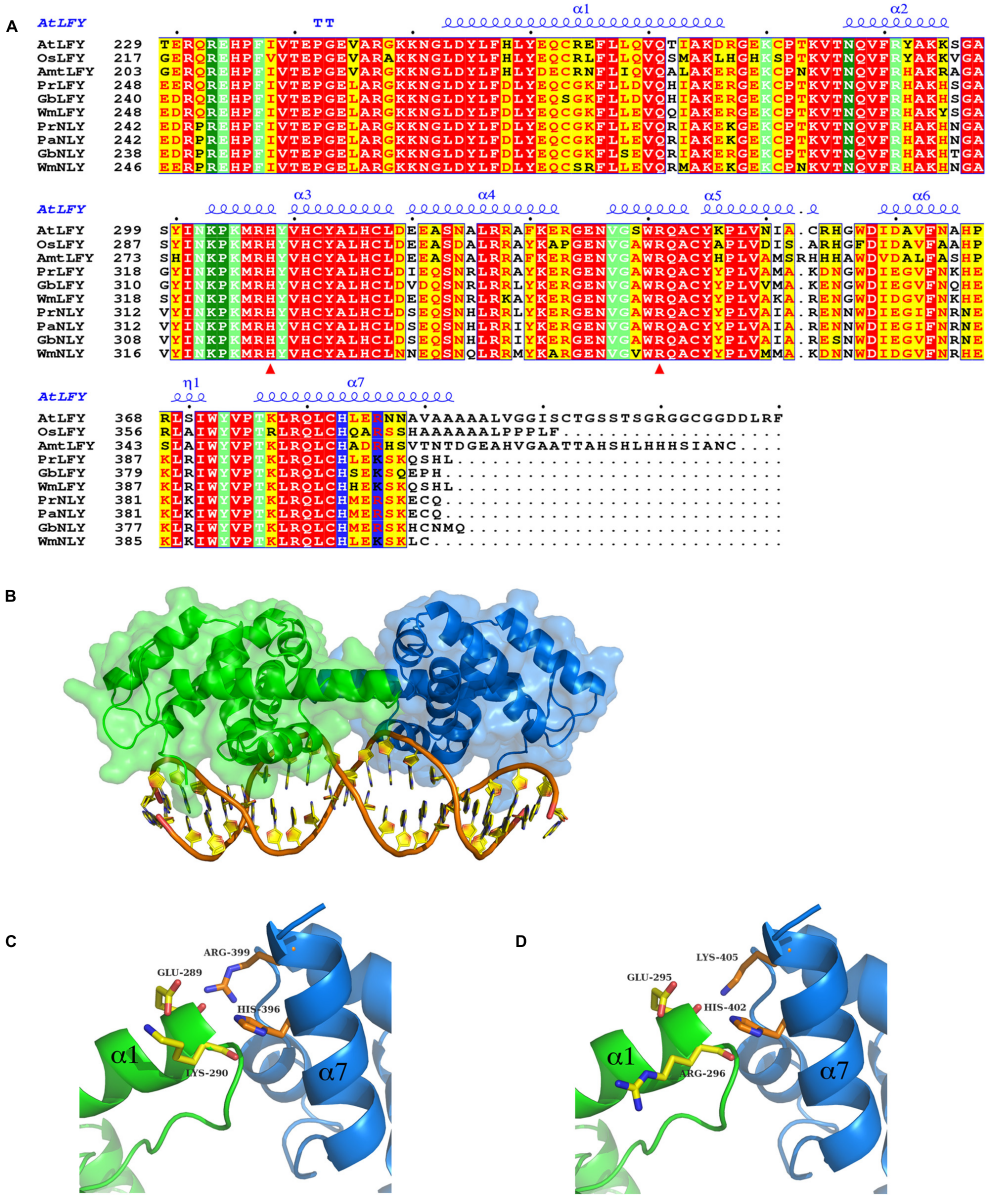
**Sequence alignment and homology models of the DNA binding domain (DBD) of LFY and NLY.** **(A)** Sequence alignment of LEAFY (LFY) and NEEDLY (NLY) DBDs. Aligned C-terminal DBD amino acid sequences of AtLFY (*A. thaliana* LFY, GenBank AED97525.1), OsLFY (*Oryza sativa japonica* LFY, RFL), AmtLFY (*Amborella trichopoda* LFY, AmboLFY), PrLFY (*Pinus radiata* LFY, PRFLL), GbLFY (*Ginkgo biloba* LFY), WmLFY (*Welwitschia mirabilis* LFY), PrNLY (*Pinus radiata* NLY), PaNLY (*Pinus armandii* NLY), GbNLY (*G. biloba* NLY), and WmNLY (*W. mirabilis* NLY). All sequences are numbered and dots mark every tenth residue above the sequences. Highly conserved regions are boxed, with similar residues represented in red against a yellow background, invariant residues represented against a red background and non-conserved residues indicated in black. The secondary structure annotation of AtLFY DBD, as derived from its three-dimensional X-ray structure (PDB 2VY1), is depicted in blue on top of the aligned sequences [alpha helices (α); strict β-turn (TT); 3_10_-helix (η)]. Residues involved in interactions with the DNA are highlighted in dark-green (direct contact with DNA bases) and light-green (sugar phosphate backbone contacts); residues involved in dimerisation are depicted in blue. Red triangles indicate residues important for determining DNA half-site specificity. The AtLFY protein sequence (AED97525.1) differs from the AtLFY sequences in Hames et al. (2008) and (Sayou et al., 2014; AAA32826) by a four residue deletion after resdiue 152 resulting in a -4 sequence shift. **(B)** Homology model of *Pinus radiata* NEEDLY (PrNLY) DBD based on AtLFY DBD X-ray structure (PDB 2VY1). Monomers are represented in green and blue as cartoons with a partial transparent surface; bound DNA is represented in orange and gold. PrNLY DBD adopts the same seven α-helix fold, contacting the DNA through both the minor and major grooves with complete conservation of all DNA-binding amino acid residues determined for AtLFY DBD. **(C)** Close-up view of the dimerisation interface of PrNLY. Monomers are colored as per **(B)** and side chain residues involved in putative hydrogen bonding interactions are shown and labeled. **(D)** Close-up view of the dimerisation interface of PrLFY. Colors and residues as per **(C)**.

**FIGURE 4:**
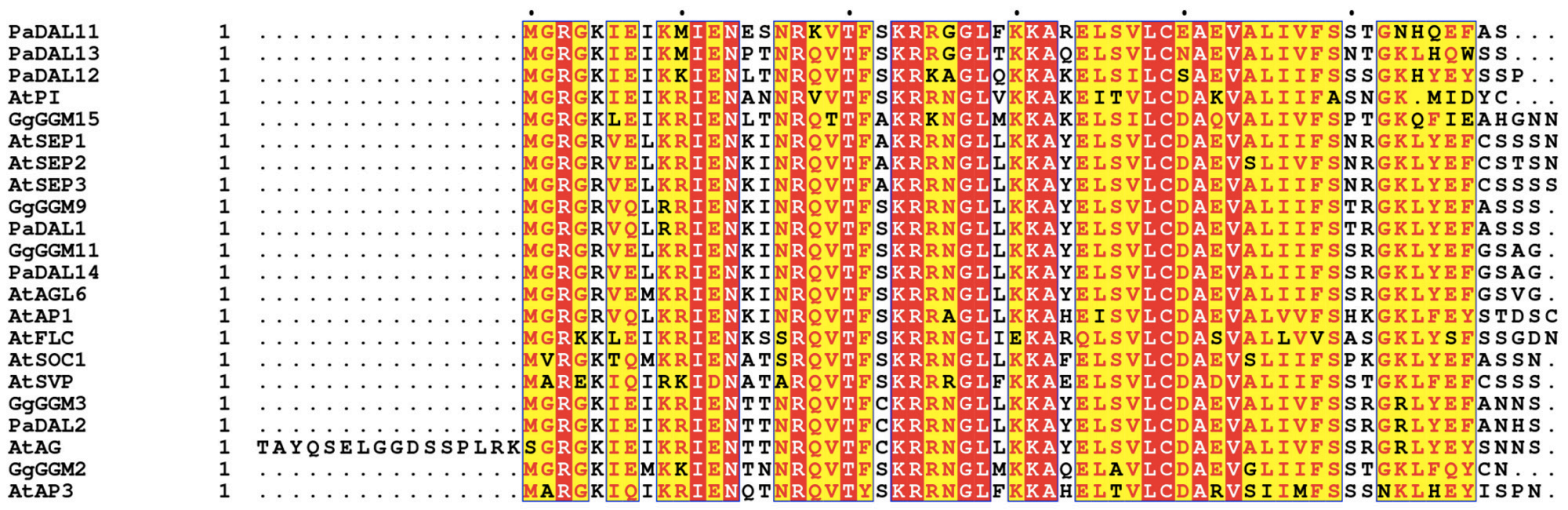
**Sequence alignment of MADS TFs M-domain.** Aligned M-domain amino acid sequences of *A. thaliana* SEP3, SEP1, SEP2, AP3, PI, AP1, AG, AGL6, SOC1, SVP, and FLC; the gymnosperm *Gnetum gnemon* GGM2, GGM15 (AP3/PI-like), GGM3 (AG-like), and GGM9, GGM11 (AGL6-like); and the gymnosperm *Picea abies* DAL11, DAL12, DAL13 (AP3/PI-like), DAL2 (AG-like), and DAL1, DAL14 (AGL6-like) proteins. Sequence numbering is indicated on the left, with every tenth residue marked by black dots above the sequences. Highly conserved regions are boxed, with similar residues represented in red against a yellow background, invariant residues represented against a red background and non-conserved residues indicated in black.

**FIGURE 5:**
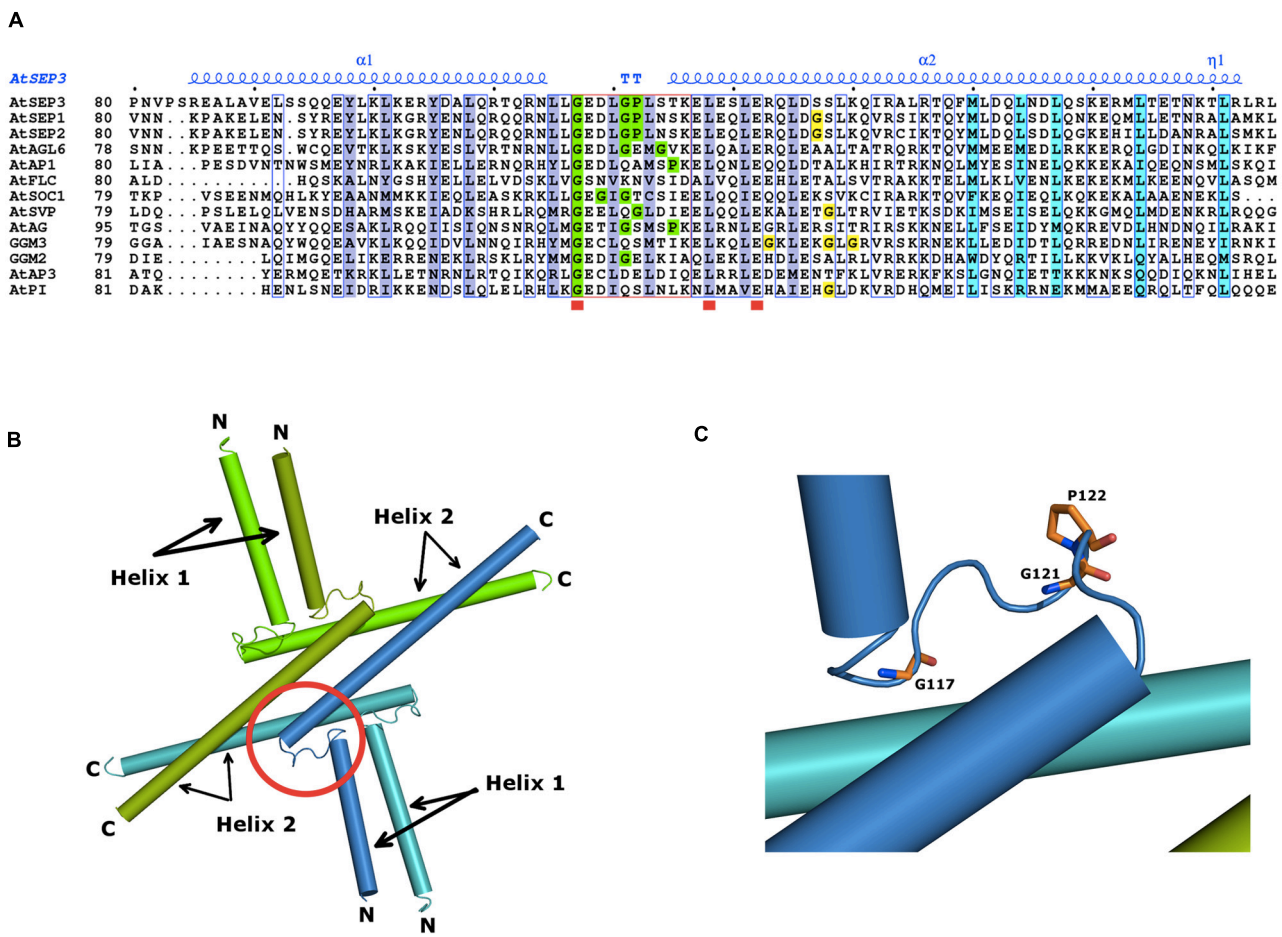
**MADS TFs oligomerisation domain.** **(A)** Sequence alignment spanning the SEP3 crystallographic structure (PDB 4OX0). All sequences are from *A. thaliana* SEP3, SEP1, SEP2, AP3, PI, AP1, AG, AGL6, SOC1, SVP, and FLC and the gymnosperm *G. gnemon* GGM2 and GGM3 proteins. Numbering is indicated at the start of the sequences and every tenth residue indicated by a black dot above the SEP3 sequence. Highly conserved regions are boxed in blue with a white background; strictly conserved residues are depicted by a red square below the sequence. The secondary structure elements of AtSEP3 K-domain (PDB 4OX0), are shown in blue above the sequences (α; TT; η). Residues involved in dimerisation and tetramerisation in SEP3 K-domain structure are highlighted in violet and cyan, respectively. The kink region between helices 1 and 2 is framed in red; Gly and Pro residues present within the kink regions are highlighted in green; Gly residues in the N-terminal region of helix 2 are highlighted in yellow. **(B)** Structure of SEP3 K-domain (PDB 4OX0). The oligomerisation domains of SEP3 are represented as cylinders; each monomer, composed of two distinct helices (helices 1 and 2), is colored uniquely in green, dark green, blue, and light blue. N and C-terminal regions are indicated. **(C)** Close-up of the SEP3 kink between helices. Glycine and proline residues are depicted as sticks colored by atom with carbons in orange.

For the LFY/NLY sequence alignments (**Figures 2** and **3**) the sequences used are as follows: AtLFY (*A. thaliana* LFY, AED97525.1), OsLFY (*Oryza sativa japonica* LFY, RFL, AHX83808.1), AmtLFY (*Amborella trichopoda* LFY, AmboLFY, AGV98899.1), PrLFY (*Pinus radiata* LFY, PRFLL, AAB51587.1), GbLFY (*Ginkgo biloba* LFY, ADD64700.1), WmLFY (*Welwitschia mirabilis* LFY, AAF23870.1), PrNLY (*P. radiata* NLY, AAB68601.1), PaNLY (*Pinus armandii* NLY, ADO33969.1), GbNLY (*G. biloba* NLY, AAF77074.1), and WmNLY (*W. mirabilis* NLY, AAD38872.1). For MADS-domain TFs sequence alignments (**Figures 4** and **5**) the sequences used are: *A. thaliana* SEP3 (AEE30503.1), SEP1 (AED92208.1), SEP2 (AEE73791.1), AP3 (AEE79216.1), PI (AED92817.1), AP1 (AEE34887.1), AG (AEE84111.1), AGL6 (AEC10582.1), SOC1 (AEC10583.1), SVP (AEC07320.1), and FLC (AED91498.1); *Gnetum gnemon* GGM2 (CAB44448.1), GGM3 (CAB44449.1), GGM15 (CAC13991.1), GGM9 (CAB44455.1), and GGM11 (CAB44457.1); *Picea abies* DAL11 (AAF18373.1), DAL12 (AAF18375.1), DAL13 (AAF18377.1), DAL2 (CAA55867.1), DAL1 (CAA56864.1), and DAL14 (AGR53802.1). Numbers indicated correspond to GenBank accession numbers.

### Homology Modeling

The homology model of the DNA-binding domain (DBD) of *Pinus radiata* NLY (PrNLY) and LFY (PrLFY) proteins were built using the SWISS-MODEL server (Arnold et al., 2006; Biasini et al., 2014, swissmodel.expasy.org). Based on the sequence alignment between PrNLY and AtLFY DBDs the PrNLY partial sequence [E242-Q404] was fed to the server, as well as the AtLFY DBD PDB structure (PDB 2VY1, GenBank accession AAA32826). The homology model of PrNLY DBD comprises residues [R246-K401]. The same procedure was applied to PrLFY for which the partial sequence [Q251-H410] was fed to the server; the PrLFY homology model comprises residues [R252-K407]. Each of the models was superimposed on the AtLFY DBD structure (Hames et al., 2008) using COOT (Emsley et al., 2010); the DNA coordinates added to the composite homology models were taken from the AtLFY structure. The cartoon model representation was made using the program Pymol (The PyMOL Molecular Graphics System, 2010).

### SEP3^(75-178)^ Mutagenesis, Expression, and Purification

SEP3^(75-178)^ construct (wild type) was cloned into the expression vector pESPRIT002 (Hart and Tarendeau, 2006; Guilligay et al., 2008) using the AatII and NotI restriction sites. The plasmid contains an N-terminal 6x-His tag followed by a TEV protease cleavage site. All mutants produced were generated using the SEP3^(75-178)^ construct as the template and using Phusion polymerase (NEB) according to the manufacturer’s protocol. The oligonucleotides used for mutagenesis are provided in **Table 1**.

**Table 1:** Oligonucleotides used for SEP3^(75-178)^ mutagenesis.

Mutant	Oligonucleotides
M150A	5′-CTCTCAGGACACAGTTTGCGCTTGACCAGCTCAAC-3′
	5′-GTTGAGCTGGTCAAGCGCAAACTGTGTCCTGAGAG-3′
L154A	5′-CAGTTTATGCTTGACCAGGCGAACGATCTTCAGAGTAAGG-3′
	5′-CCTTACTCTGAAGATCGTTCGCCTGGTCAAGCATAAACTG-3′
L171A	5′-CTGACTGAGACAAATAAAACTGCAAGACTAAGGTTAGCTGATGG-3′
	5′-CCATCAGCTAACCTTAGTCTTGCAGTTTTATTTGTCTCAGTCAG-3′
Splice^Δ161-174^	5′-CCAGCTCAACGATCTTCAGAGTAAGCTAGCTGATGGATGAGAGACAAATAAAACTCTAAGACTAAGG-3′ (forward)
	5′-CCTTAGTCTTAGAGTTTTATTTGTCTCTCATCCATCAGCTAGCTTACTCTGAAGATCGTTGAGCTGG-3′ (reverse)
Y98K	5′-CTTAGTAGCCAGCAGGAGAAGCTCAAGCTTAAGGAGCG-3′
	5′-CGCTCCTTAAGCTTGAGCTTCTCCTGCTGGCTACTAAG-3′
Y105N	5′-CTCAAGCTTAAGGAGCGTAATGACGCCTTACAAAGAACCC-3′
	5′-GGGTTCTTTGTAAGGCGTCATTACGCTCCTTAAGCTTGAG-3′
L115R	5′-CAAAGAACCCAAAGGAATAGGTTGGGAGAAGATCTTGGACCTC-3′
	5′-GAGGTCCAAGATCTTCTCCCAACCTATTCCTTTGGGTTCTTTG-3′
L131V	5′-CTAAGTACAAAGGAGCTTGAGTCAGTTGAGAGACAGCTTGATTC-3′
	5′-GAATCAAGCTGTCTCTCAACTGACTCAAGCTCCTTTGTACTTAG-3′
L135M	5′-CTTGAGTCACTTGAGAGACAGATGGATTCTTCCTTGAAGC-3′
	5′-GCTTCAAGGAAGAATCCATCTGTCTCTCAAGTGACTCAAG-3′
L135A	5′-GTCACTTGAGAGACAGGCTGATTCTTCCTTGAAGC-3′
	5′-GCTTCAAGGAAGAATCAGCCTGTCTCTCAAGTGAC-3′


SEP3^(75-178)^ and all the tetramerisation mutant constructs were overproduced in *Escherichia coli* BL21 (DE3) CodonPlusRIL (Agilent Technologies; Puranik et al., 2014); all dimerisation mutant constructs were overproduced in *E. coli* Rosetta2 (DE3) pLysS cells (this study). Cells were grown at 37°C in Luria-Bertani (LB) culture medium supplemented with kanamycin (50 mg/mL) and chloramphenicol (37 mg/mL), until an OD_600_ of 0.7–0.8 was reached. At this point, protein expression was induced by addition of 1 mM isopropyl-β-D-galactopyranoside (IPTG) and the temperature reduced to 20°C; expression was continued for 16 h (overnight). Cells were harvested by centrifugation at 6000 rpm for 30 min at 4°C and then resuspended in Buffer A [30 mM Tris pH 8.0, 300 mM NaCl, 5% (v/v) glycerol, 2 mM TCEP] to which benzonase (Sigma) and protease inhibitors (Roche EDTA-free) were added. Cells were disrupted by sonication, followed by centrifugation at 25000 rpm for 40 min at 4°C, to remove cell debris. The cell lysate was then passed onto a column containing 1 mL of Ni-Sepharose High-Performance resin (GE-Healthcare), previously equilibrated with Buffer A. Bound protein was washed in two steps: high salt (30 mM Tris pH 8.0, 1 M NaCl, 5% glycerol, 2 mM TCEP) and low imidazole concentration (buffer A + 20 mM Imidazole); and subsequently eluted with Buffer B (30 mM Tris pH 8.0, 300 mM NaCl, 5% glycerol, 250 mM Imidazole, 2 mM TCEP). Fractions of interest were pooled and dialysed overnight at 4°C, against Buffer A and in the presence of 2% (w/w) TEV protease, in order to cleave the 6xHis tag. The protein sample was passed over the same 1 mL Ni-Sepharose column, in order to deplete the His-tagged TEV protease and remove uncut protein from the cleaved protein sample. The purified protein was then concentrated and applied onto a size exclusion Superdex 200 10/300 GL column (GE Healthcare), pre-equilibrated with Buffer A. SEP3^(75-178)^ and all mutants were purified following this same protocol.

### EMSA Experiments

AG, SEP3 full length wild type, and SEP3 mutants (L171A, L115R, SEP3^ΔC^) were cloned into a pSPUTK plasmid and used for *in vitro* transcription translation (Promega SP6 High Yield Expression System). SEP3^ΔC^ contains residues 1–160 with a – LADG-stop terminating sequence corresponding to a complete truncation of the C-terminal domain. Protein expression was performed as per the manufacturer’s protocol and used without further purification. *SOC1* promoter DNA (121 bp *SOC1* specific DNA) comprising two CArG boxes was used as per (Kaufmann et al., 2009). Two *SOC1* promoter DNA fragments were generated with either the first or second CArG box mutated. Mutations were generated using a 1 kb *SOC1* promoter DNA as the template (inserted into pCR blunt vector) and using Phusion polymerase (NEB) according to the manufacturer’s protocol. CArG-box 1 was mutated with the forward primer 5′-CGTGTCTAAAGAGGCATTTGACATATGACGTCCCTCGGATTACTAAAG-3′ and the reverse primer 5′-CTTTAGTAATCCGAGGGACGTCATATGTCAAATGCCTCTTTAGACACG (CArG-box 1 mutation is underlined); and CArG-box 2 mutated with the forward primer 5′-GTGGCACCAAAAAAATATACATATGACGAGATAAAATTGTTAATCG-3′ and the reverse primer 5′- CGATTAACAATTTTATCTCGTCATATGTATATTTTTTTGGTGCCAC-3′ (CArG-box 2 mutation is underlined). Final 145 bp mutated *SOC1* DNA fragments were then PCR amplified using the primers 5′-CTAAAGAGGCATTTGACATATGACGTCCCTCG (fwd) and 5′-GATTAACAATT TTATCTCCAAAAAAGGATATTTTTTTGG (rev) for CArG-box 1, and 5′-CTAAAGAGGCATTTGCTATTTTTGGTCCCTCG (fwd) and 5′-GATTAACAATTTTA TCTCGTCATATGTATATTTTTTTGG (rev) for CArG-box 2 mutated DNAs, respectively. *SOC1* DNA labeled with DY-682 (Dyomics GmbH, wild type) or Cy5 (Eurofins, mutated CArG-boxes) was used at a concentration of approximately 5–10 nM for all reactions in a protein binding buffer containing 7 mM HEPES, pH 7.0, 1 mM BSA, 1 mM EDTA, 1 mM DTT, 2.5% CHAPS, 6% glycerol, 0.06 mg/ml salmon sperm DNA, 1.3 mM spermidine. 4 μl of TnT protein mix was added directly without purification to the binding buffer to a final volume of 20 μl.

### AG Expression and Purification

AG^(74-173)^ was cloned into a pESPRIT002 vector using NotI and AatII restriction sites. The construct contained an N-terminal TEV protease cleavable poly-histidine tag (Hart and Tarendeau, 2006; Guilligay et al., 2008). The protein was overexpressed in *E. coli* BL21 Star (DE3)pLysS cells (Life Technologies). Cells were grown in Luria Bertani medium in the presence of 50 mg/ml kanamycin and 35 mg/ml chloramphenicol at 37°C and 180 rpm to an optical density A_600_ = 0.8 after which time the temperature was lowered to 20°C and 0.2 mM isopropyl β-D-1-thiogalactopyranoside (IPTG) was added for induction. After 16 h, the cells were harvested by centrifugation at 6000 rpm and 4°C for 15 min and resuspended in lysis buffer containing 30 mM Tris pH 8.0, 300 mM NaCl, 1 mM TCEP, 5%(v/v) glycerol, 20%(w/v) sucrose and 1x protease inhibitors (Roche EDTA-free). Cells were lysed by sonication and the insoluble fraction pelleted by centrifugation at 25000 rpm and 4°C for 30 min. The pellet was resuspended in denaturation buffer [30 mM Tris pH 8.0, 300 mM NaCl, 1 mM TCEP, 5% (v/v) glycerol, 8 M Urea] and incubated for 1 h at room temperature. The solubilized fraction was applied to a 5 ml Ni-NTA column pre-equilibrated with denaturation buffer, followed by a wash with 10 CV of wash buffer (30 mM Tris pH 8.0, 300 mM NaCl, 1 mM TCEP, 5% glycerol, 8 M Urea, 30 mM imidazole) and eluted with 3 CV of elution buffer (30 mM Tris pH 8.0, 300 mM NaCl, 1 mM TCEP, 5% glycerol, 8 M Urea, 300 mM Imidazole). The eluted fraction was dialysed step-wise against 6, 4, and 2 M urea plus 30 mM Tris pH 8.0, 300 mM NaCl, 1 mM TCEP, 5% glycerol. After the final dialysis step, the protein was applied to a size exclusion chromatography column (Superdex 75 10/300 GL, GE Healthcare) pre-equilibrated with gel filtration buffer [30 mM Tris pH 8.0, 300 mM NaCl, 1 mM TCEP, 5% (v/v) glycerol]. The purity of the final fractions was assessed using SDS-PAGE. Fractions of interest were pooled and incubated overnight with TEV protease to remove the poly-histidine tag. After depletion of TEV and uncleaved protein over a 5 ml Ni-NTA column, the cleaved AG^(74-173)^ was loaded onto a Superdex S75 10/300 GL column as a final purification step and the fractions of interest pooled and concentrated to approximately 4 mg/ml for SAXS studies.

### SAXS Data Collection

An on-line hplc system (Viscotek, Malvern Instruments) was attached directly to the sample inlet valve of the BM29 sample changer (European Synchrotron Radiation Facility, bioSAXS bending magnet beamline 29; Pernot et al., 2013; Round et al., 2015). The protein sample (50 μl) was injected onto the column (Superdex 75 3.2/300 PC, GE Healthcare) after column equilibration. Buffers were degassed prior to the run and a flow rate of 0.1 ml/min at room temperature was used. Buffers used were as described above. All data from the run was collected using a sample to detector (Pilatus 1 M Dectris) distance of 2.86 m corresponding to an s range of 0.04–4.9 nm^-1^. Approximately 1800 frames (1 frame/sec) per hplc run were collected. Initial data processing was performed automatically using the EDNA pipeline (Incardona et al., 2009), generating radially integrated, calibrated, and normalized 1-D profiles for each frame. All frames were compared to the initial frame and matching frames were merged to create the reference buffer. Any subsequent frames which differed from the reference buffer were subtracted and then processed within the EDNA pipeline using tools from the EMBL-HH ATSAS suite (Petoukhov and Svergun, 2007). The invariants calculated by the ATSAS autoRg tool were used to select a subset of frames from the peak scattering intensity. The 49 frames corresponding to the highest protein concentration were merged manually and used for all further data processing and model fitting. Molecular weight for the protein was estimated based on the correlated volume (Rambo and Tainer, 2013). The approximate molecular weight was 21 kDa, corresponding to a dimer. The volume of 36 nm^3^ was calculated using the GNOM interface of the cross platform version of PRIMUS for the ATSAS software suite.

### AG Model Fitting

Homology models for AG^(74-173)^ were generated based on the SEP3 structure (PDB 4OX0; Puranik et al., 2014). For the elongated conformation, the kink between helices 1 and 2 was removed, the helices superposed and residues corresponding to the flexible region between the helices built in manually using COOT with idealized geometry and no secondary structure restraints. The model for the bent conformation was generated by threading the sequence of AG^(74-173)^ directly onto the SEP3 dimer (4OX0). Structures corresponding to two different dimer conformations (bent and elongated) were used to calculate theoretical scattering curves. These curves were compared with the experimental data using CRYSOL (Svergun et al., 1995).

## Results and Discussion

### LEAFY and NEEDLY Structure and Function-Homology Modeling of the DBDs

The angiosperm *LFY* gene is most often found as a single copy (Brunkard et al., 2015), however, gymnosperms possess two paralogous genes- *LFY* and *NLY*, born from an ancient duplication which occurred before the divergence of the angiosperm and extant gymnosperm lineages. Examination of the genomes of gymnosperms available through the 1000 plant genomes project as well as all partial deposited sequences reveals that *LFY* and *NLY* are present in all gymnosperm genomes characterized to date, with the exception of the genus *Gnetum* where *NLY* is absent. The proteins the *LFY* and *NLY* genes encode comprise two distinct domains, a partially conserved N-terminal domain (**Figure 2**) important for complex formation and a highly conserved C-terminal DBD (70% sequence identity between AtLFY and WmNLY, for example; **Figure 3A**), with connecting regions presenting a higher degree of variability. In order to probe the function of these proteins, we first aligned the DBDs of LFY and NLY using ClustalW in order to assess conservation of DNA-binding specificity (**Figure 3A**). We observed that the DBDs of LFY and NLY are highly conserved in all seed plants based on sequence alignment. To investigate any potential changes in quaternary structure or putative alterations in the DNA-binding interface, the crystal structure of the DBD of LFY from *A. thaliana* (Hames et al., 2008) was used as a homology model to generate 3D models of the DBDs of gymnosperm LFY and NLY (**Figure 3B**) using SWISS-MODEL with default parameters. Comparison of the primary sequences with secondary, tertiary and quaternary structure derived from the crystallographic data revealed that the DBDs are structurally identical and all amino acids involved in direct contacts with DNA are completely conserved between angiosperm LFY (aLFY), gymnosperm LFY (gLFY), and NLY. In addition, the dimerisation interface recently described as a key component in DNA binding specificity (Sayou et al., 2014) is also highly conserved between gymnosperms and angiosperms as shown in **Figure 3**. However, while AtLFY His383 is almost completely conserved in both angiosperms and gymnosperms, based on all available sequence data, the residue at position AtLFY 386 varies as either an arginine in aLFY and NLY (Arg399 in PrNLY, **Figure 3C**) or by substitution as a lysine in gLFY (Lys405 in PrLFY, **Figure 3D**). Arginine and lysine fulfill similar structural roles and can substitute for one another due to the conserved positive charge and hydrogen bonding ability of the primary 𝜀-amine and guanidine group for lysine and arginine, respectively (Sokalingam et al., 2012). However the higher pKa and longer size of the arginine side chain may affect the hydrogen bonding interaction with the carbonyl oxygen of residue 276 (AtLFY; residue 289, PrNLY, **Figure 3C**) and cannot be ruled out as affecting dimer stability, and possibly conformation (relative positioning of the monomers). Overall, the high degree of sequence identity between the DBD of aLFY, gLFY, and NLY implies a likely conserved recognition of cognate DNA sequences. Recent studies by Sayou et al. (2014) have demonstrated the evolutionary trajectory of LFY from green algae to moss to angiosperms based on structural and biochemical studies of several DBDs including those of *Klebsormidium subtile* LFY (algae), *Physcomitrella patens* LFY (moss), and *Arabidopsis* LFY (Sayou et al., 2014). The distantly related LFY from algae, moss and angiosperms were shown to bind different DNA motifs due to small changes in the LFY dimerisation interface, as well as in two other key amino acids (AtLFY His308 and Arg341) that determine the DNA half-site sequence recognized (**Figure 3A**), as previously determined through a combination of structural and SELEX experiments (Sayou et al., 2014). However, the SELEX motif for the DBD of gymnosperm *G. biloba* LFY (GbLFY) is almost identical to the SELEX motif for the DBD of AtLFY (Sayou et al., 2014). As the dimerisation interface and all residues directly contacting the DNA are highly conserved in angiosperms and gymnosperms for aLFY, gLFY and NLY, this suggests the proteins are able to bind the same or very similar DNA motifs. Thus, it is probable that the DBD of LFY/NLY in higher plants became fixed, with conservation of DNA binding and dimerisation motifs. While bound DNA sequences and DNA binding matrices are available for LFY from *A. thaliana* based on multiple ChIP-seq and SELEX studies, no such data is available for gLFY or NLY with the exception of the GbLFY motif. Additional data would be important to confirm that there are no subtle allosteric effects that may tune the DNA binding specificity of these different paralogs, a possibility that cannot be excluded based on available data.

### Functional Implications of Complex Formation- the Role of the N-terminal Domain in LFY and NLY Function

Interestingly, functional studies do not show full complementation of a *lfy* mutant in *A. thaliana* by either *gLFY* (from *P. radiata*) or *NLY* (from *W. mirabilis*; Maizel et al., 2005). If the DBDs are able to recognize the same DNA sequences, why do gLFY and NLY less efficiently complement the *Arabidopsis lfy* mutant? One explanation relies on complex formation with ternary factors that may tune DNA binding specificity, for example through multi-site binding of different adjacent *cis*-elements. This suggests that differences in target gene regulation for aLFY, gLFY, and NLY likely rests on the structure and function of the N-terminal non-conserved regions of the LFY and NLY proteins. While the DBDs are virtually identical, the sequence conservation in the N-terminal regions of aLFY, gLFY, and NLY is much lower (**Figures 2** and **3A**).

The ability to interact with specific partners and form different ternary complexes changes the ability of a TF to regulate downstream genes. By retaining the core DBD and the essential DNA-binding functionality, the N-terminal region of the protein could vary, thus leading to relatively smooth changes in gene regulation over the course of evolution by simply tuning the interactions with ternary partners and thus modulating interactions with cognate DNA without requiring altering the DBD itself. The N-terminal ∼200 residues of LFY have been shown to be important for dimerisation (Siriwardana and Lamb, 2012) and can possibly play a role in the formation of higher order complexes with chromatin remodelers and other TFs (Wu et al., 2012). Indeed, unfolded, flexible loops, and low-complexity regions exhibit greater variability and tolerance for mutations, as they do not affect the overall fold of the macromolecule. In addition, these regions often have important functions and act as protein–protein interaction surfaces (Dyson and Wright, 2005). While alpha-helices are relatively disfavored as protein–protein interaction interfaces, exposed beta strands, hydrophobic patches and long loops are more likely to play a role in complex formation (Jones and Thornton, 1996; Neuvirth et al., 2004). These structural motifs are able to create relatively planar surfaces which are often correlated with protein–protein interactions (Hoskins et al., 2006). Few mapping studies of LFY have been performed and only a small number of interaction surfaces with partner proteins have been determined (Chae et al., 2008; Souer et al., 2008; Pastore et al., 2011; Siriwardana and Lamb, 2012; Wu et al., 2012). From the limited data available, however, it seems that several partners interact with the N-terminal region of the protein (Souer et al., 2008; Siriwardana and Lamb, 2012). Structural characterization of the N-terminal domain of LFY would allow determining whether its properties might have changed during evolution.

Due to the loss of NLY in the angiosperm lineage, aLFY likely assumed additional functions, fusing the functionality of NLY, a key regulator of female organ development, and gLFY, an important primary regulator of male cone development, into one fully competent regulator of plant reproduction. As has been recently shown for several conifers (*Picea abies, Podocarpus reichei*, and *Taxus globosa*), LFY and NLY have overlapping expression patterns (Vazquez-Lobo et al., 2007; Carlsbecker et al., 2013). This would mitigate any deleterious effects of NLY loss during the gymnosperm/angiosperm split by allowing more facile compensation for NLY function by LFY, as LFY was already present in the same tissues, possessed the same DBD, and likely recognized very similar cognate DNA sequences. Thus, aLFY compensation for NLY/gLFY during reproductive development would not necessitate extensive reprogramming of LFY expression patterns nor require any changes to the gene coding sequence of the DBD, important factors in the successful compensation due to gene loss of NLY in the angiosperm lineage.

### MADS-Domain TFs and Their Role in Floral Organ Development

The homeotic class A-E MADS-box genes direct the specification of all the floral organs and as such are central players in flower evolution and development. In gymnosperms, orthologs to the B and C class MADS-box genes (*AP3/PI* and *AG* in *Arabidopsis*) are also present and play important roles in male and female organ development. While the MADS-box gene family has expanded in all land plants, this is most striking in angiosperms due to extensive duplication events giving rise to the class E *SEPALLATA* genes, which are not present in extant gymnosperms (Zahn et al., 2005a). The SEPALLATA (SEP) proteins have acquired new functionality and act as mediators of interactions between class A, B, and C MADS-domain TFs as shown by yeast two and three hybrid studies, EMSA experiments and *in vivo* studies (Pelaz et al., 2000; Honma and Goto, 2001; Kaufmann et al., 2005; Malcomber and Kellogg, 2005; Theissen and Melzer, 2007; Immink et al., 2009; Mendes et al., 2013). The SEP proteins form heteromeric complexes with other MADS TFs and all putative floral organ-specifying tetrameric MADS complexes contain at least one SEP protein leading to the specification of the different floral organs (Theissen, 2001; Theissen and Saedler, 2001). Indeed, *sep123* mutants are sterile and unable to produce male or female organs, with the flower converted to a collection of sepaloid-like structures, illustrating the requirement of the SEP proteins for proper reproductive organ formation (Pelaz et al., 2000). Examination of the B and C class MADS TFs in gymnosperms such as *G. gnemon* suggests that tetramerisation can occur and is necessary for male and female organ development (**Figure 1**). This tetramerisation takes place without the obligatory mediation of the class E-like AGL6 proteins (Wang et al., 2010). However, angiosperms are dependent on the class E SEPs for tetramer formation, as the B and C class TFs have lost their ability to directly interact. Current hypothesis suggest that the changing interaction patterns of the MADS TFs, in particular the requirement of the SEPs to mediate tetramer formation in angiosperms is at the nexus of flower origins (Melzer et al., 2010; Wang et al., 2010). The evolution of the bisexual flower thus requires an understanding, at the protein level, of the MADS TFs, particularly how the SEPs are able to mediate the formation of tetrameric complexes which are critical to the development of all the floral organs.

Our recent crystallographic data of the oligomerisation domain of SEP3 (Puranik et al., 2014), together with mutagenesis studies, sequence alignments and biophysical characterization of the C-class MADS TF AGAMOUS (this study) help to explain the molecular function of the MADS TFs and contribute to our understanding of flower evolution. All MADS homeotic TFs are characterized by a four domain arrangement consisting of a highly conserved DBD “M” domain (∼60 amino acid MADS domain, **Figure 4**), an “I” domain (linker Intervening domain) important for dimerisation, a “K” domain (alpha helical Keratin-like domain) critical for dimerisation and tetramerisation, and a “C” domain (highly variable C-terminal domain) important for different functions including transactivation and higher order complex formation (Kaufmann et al., 2005). Based on the crystal structure of a portion of the I and the full K domain of SEP3 (Puranik et al., 2014) and extensive mutagenesis studies, the dimerisation and tetramerisation interfaces of the MADS-domain TFs can be mapped at the amino acid level (**Figures 5A,B**). Different amino acids along the dimer and tetramer interface were targeted for mutagenesis studies in order to probe the mechanisms of oligomerisation and stability (**Table 2**). Mutation of any residue making a direct contact with its partner along the dimer (Leu115, Leu131, Leu135, Tyr98, Tyr105; this study) or tetramer (Met150, Leu154, Leu171; Puranik et al., 2014) interface in SEP3 had a striking effect on oligomerisation, with even a single point mutation greatly destabilizing the complex as determined by size exclusion chromatography and comparison with the wild type protein. This suggests that subtle differences in the amino acids at the dimerisation and tetramerisation surface will shift the oligomerisation equilibrium to favor certain complexes when multiple MADS TFs are present. Examining structure based sequence alignments for the homeotic MADS-domain TFs demonstrates a conservation of hydrophobic residues at the oligomerisation interface, but the size and shape of these residues varies, which will help mediate protein–protein interactions (**Figure 5A**).

**Table 2:** Effect of point mutations on SEP3 oligomerisation.

	SEP3^(75-178)^ constructs	Oligomerisation state
Tetramerisation interface	Wild type	**Tetramer**/dimer
(Puranik et al., 2014)	M150A	**Dimer**
	L154A	**Dimer**
	L171A	**Dimer**
	Splice^Δ161-174^	**Dimer**
Dimerisation interface	Y98K	**Dimer**/monomer
(this study)	Y105N	**Tetramer**/monomer
	L115R	Unstable complex
	L131V	Unstable complex
	L135M	**Dimer**/monomer
	L135A	Unstable complex


*Point mutations targetting the highly conserved residues involved in the putative dimerisation and tetramerisation interface of SEP3 were chosen for mutational analysis. Oligomeric state was determined by size exclusion chromatography. Where two states exist, the predominant species is marked in bold. “Unstable complex” is used to denote a complex mixture of species between monomeric, dimeric, and tetrameric states with no predominance for a particular oligomerisation state.*

**FIGURE 6:**
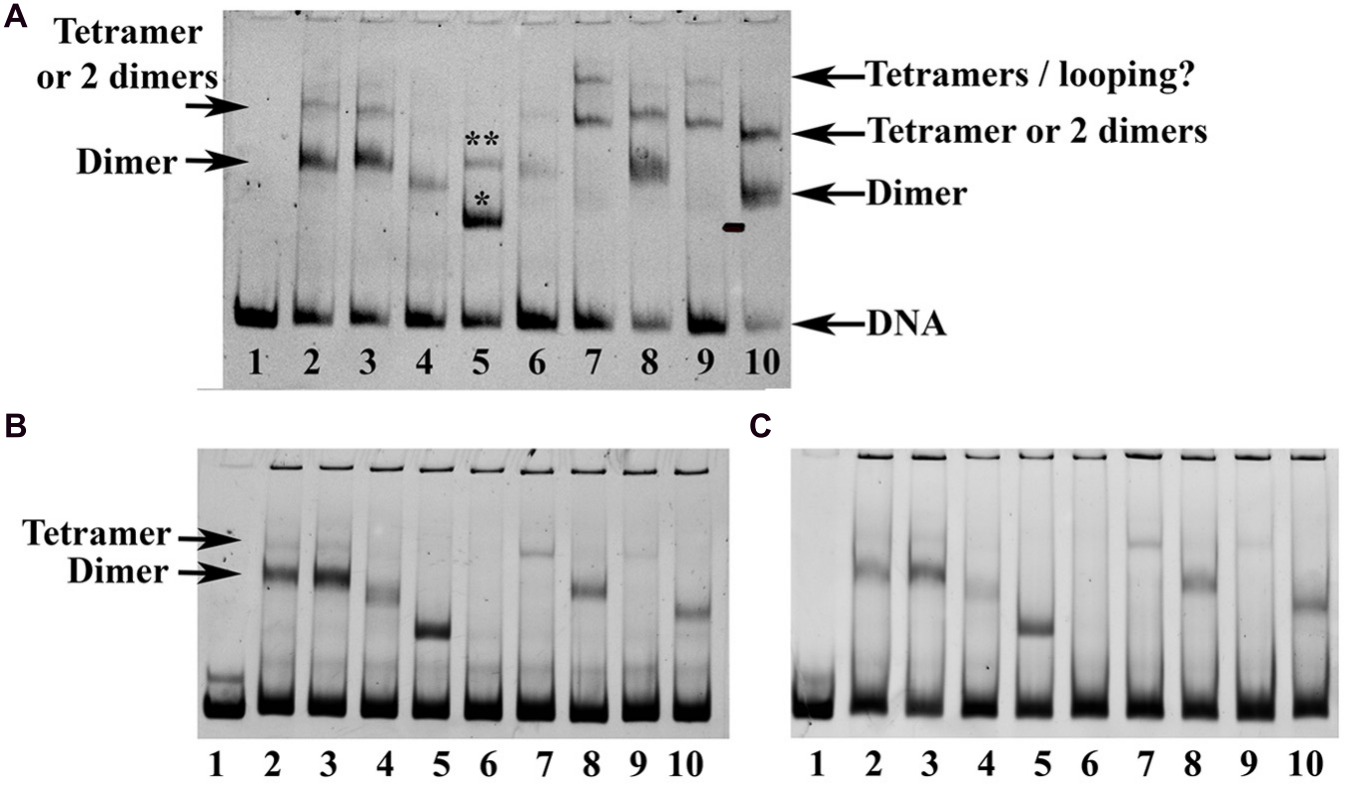
**Electrophoretic mobility shift assay (EMSA) for SEP3 and AG.** **(A)** Comparison of the oligomerisation state of SEP3 wild type, dimerisation and tetramerisation mutants with AG using a 121 bp DNA fragment of the SOC1 promoter comprising two CArG boxes. Lane 1 corresponds to DNA alone, lane 2 SEP3 wild type, lane 3 SEP3^L171A^, lane 4 SEP3^L115R^, lane 5 SEP3^ΔC^ truncation mutant, lane 6 AG, lane 7 SEP3 + AG, lane 8 SEP3^L171A^ + AG, lane 9 SEP3^L115R^ + AG, lane 10 SEP3^ΔC^ + AG. Putative tetramer and dimer are indicated by arrows. The truncation mutant in lane 5 likely has one or two dimers bound to DNA as indicated above the bands by a single (one dimer bound) or double (two dimers bound) asterix. A faint highly retarded band corresponding to bound tetramers or tetramer-induced DNA-looping was noted in lanes 7 and 9 and indicated. **(B)** EMSAs run with either CArG box 1 (left) or CArG box 2 (right) mutated (see Materials and Methods). Proteins are as per **(A)**. A faint band for SEP3 wild type and SEP3^L171A^ was noted running as per the tetramer band in **(A)**, suggesting homotetramer formation on a single CArG box site. All proteins were produced via *in vitro* transcription translation using equivalent amounts of template DNA and equivolumes of the reaction mixture were added to the final binding reaction. DNA was approximately 5–10 nM and labeled with DY-682 **(A)** or Cy5 **(B)** for imaging.

Based on the structure of the SEP3 homotetramer and mutagenesis studies, we probed the formation of hetero-oligomers using electrophoretic mobility shift assays (**Figure 6**). EMSA experiments and identification of putative complexes were performed according to previously published work (Smaczniak et al., 2012). SEP3 dimerisation and tetramerisation mutants were tested for DNA binding with AG, all expressed using *in vitro* transcription translation due to the difficulties in producing folded full length MADS TFs using standard recombinant bacterial expression. Sufficient heterodimers and tetramers were produced and a gel shift assay was performed using DNA corresponding to the *SOC1* promoter containing two CArG-box MADS TF binding sites (**Figure 6A**) and the *SOC1* promoter sequence with either the first or the second CArG-box mutated (**Figure 6B**). A SEP3 dimerisation-interface mutant, SEP3^L115R^, was dramatically impaired in its ability to oligomerise based on studies of the K-domain alone (**Table 2**), however, it was able to bind DNA as a homodimer and heterotetramer with AG, albeit with less efficiency than the wild type SEP3. The SEP3^L115R^ mutant was designed to mimic the sequence of AtAP3, which is unable to form homodimers but still retains the ability to interact with partners such as AtPI (Riechmann et al., 1996; Winter et al., 2002; Yang et al., 2003). Both AtPI and AtAG have a leucine residue at position 115, which is likely able to accommodate the arginine side chain during hetero-oligomer formation. AGAMOUS alone exhibited poor binding to the *SOC1* DNA due either to lower protein production in the *in vitro* transcription translation reaction or non-optimal sequences of the DNA, however, AG heterodimers with SEP3 were able to bind the *SOC1* sequences, suggesting differences in sequence specificity are important for AG homo and heteromer DNA binding interactions. Tetramerisation interface mutants SEP3^L171A^ and a truncation mutant (SEP3^ΔC^) showed greatly impaired heterotetramerisation with AG, as expected. Altogether, these data provide strong evidence that the homotetramerisation interface observed in the crystal structure of SEP3 is conserved in the formation of heterotetramers.

Changes in the tetramerisation interface in SEP partner MADS proteins also has an effect on oligomer formation. For example, studies of the C-class genes *PLENA (PLE)* and *FARINELLI (FAR)* from *A. majus* demonstrate that a single amino acid change was responsible for neofunctionality of these duplicated genes with *FAR* able to specify only male organs and *PLE* able to specify both male and female organs in a complementation assay in *Arabidopsis*. This activity was due to a single amino acid insertion in the K domain that altered the oligomerisation capabilities of PLE and FAR with the SEPALLATA proteins (Airoldi et al., 2010). An amino acid insertion shifts the hydrophobic pattern of all amino acids in the leucine zipper tetramerisation interface, thus modulating the hydrophobic protein–protein interface of the putative tetrameric complexes formed by PLE and FAR with their SEP partners.

In addition to the hydrophobic dimer and tetramer interface acting as a driver for oligomerisation, a key component of the MADS TFs oligomerisation propensity is the presence of a kink in between alpha helices 1 and 2 of the K domain (**Figures 5B,C**). Based on sequence alignments of the MADS homeotic TFs, this kink region is highly variable in the family with a tight turn predicted for SEP1, SEP2 and SEP3 due to the presence of a GlyPro motif (**Figure 5A**). Prolines act as “breakers” in an alpha helix due to their inability to form the appropriate hydrogen bonding interactions between the carbonyl backbone and amide proton due to the presence of the proline side chain. Glycine residues exhibit a high degree of conformational flexibility and have been shown to lead to kinks in alpha helices in soluble and membrane proteins (Wilman et al., 2014). These residues result in the formation of a tight turn and, in the case of SEP3, an approximately 90° bend between alpha helices 1 and 2 (**Figure 5B**). Examination of the sequences of other MADS TFs show scattered glycine and/or proline residues between helices 1 and 2, but not a conservation of the GlyPro motif observed in SEP1, SEP2 and SEP3. In order to investigate whether the presence of a GlyPro motif is required for complete opening of helices 1 and 2, we recombinantly overexpressed and purified the K-domain of AG.

**FIGURE 7:**
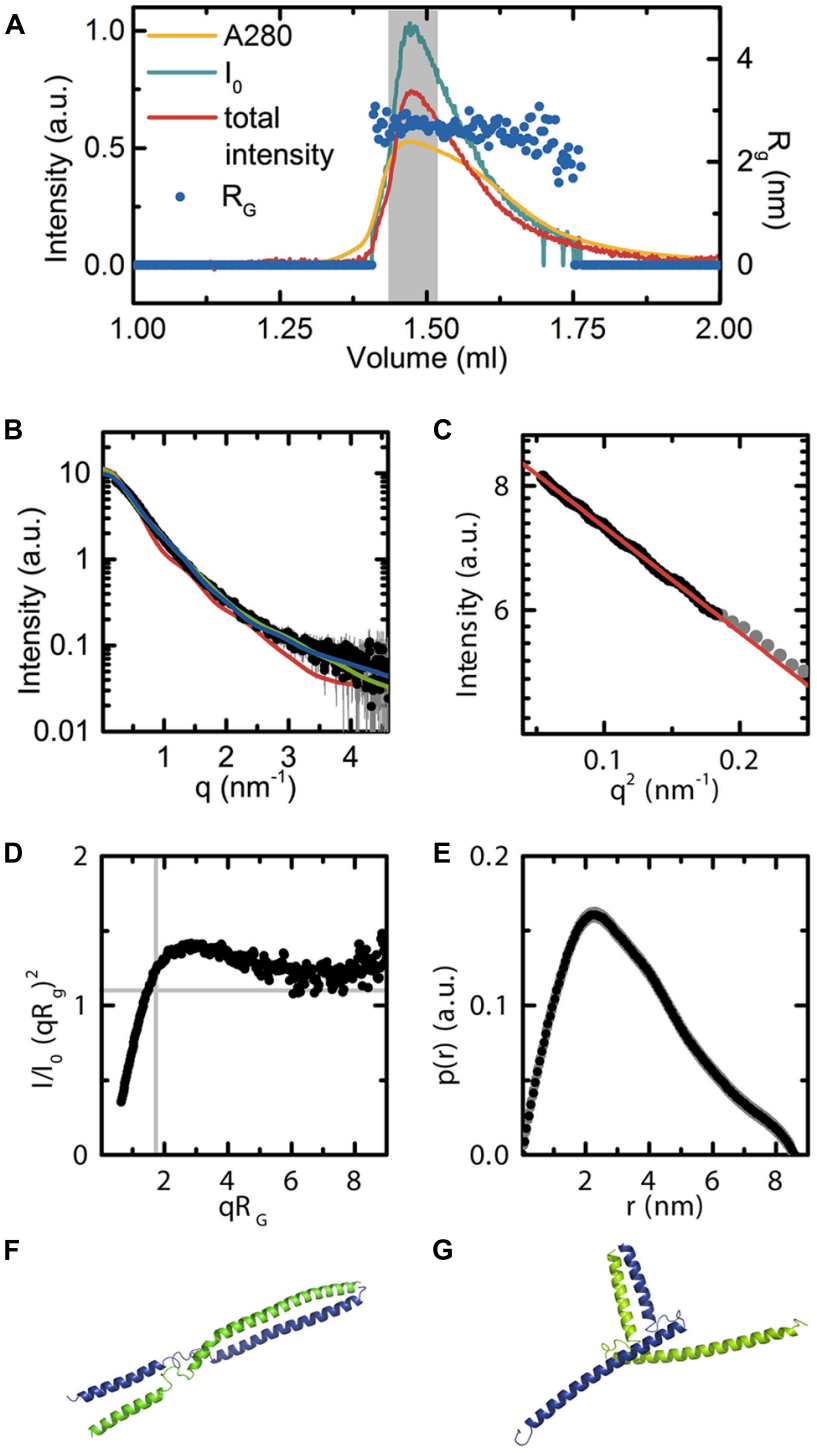
**Small angle X-ray scattering data for AG^(74-173)^.** **(A)** Experimental data showing the UV absorbance (yellow), X-ray scattering intensity (blue), and total intensity after buffer subtraction (red) for all collected frames. The radius of gyration across frames corresponding to the eluted peak are shown as dark blue dots (R_g_ = 2.7). The broad peak and slight variation in R_g_ corresponds to conformational flexibility of the protein in solution. The region of frames integrated for further analysis are highlighted in gray. Axes are as labeled. **(B)** Scattering curve in black for the integrated frames. CRYSOL fits for the bent and elongated dimer conformations, as well as the tetrameric SEP3 structure. Chi squared values were 5.6 for the elongated model (blue curve), 2.0 for the bent model (green curve) and 38.4 for the tetrameric model (red curve). **(C)** Close-up of the Guinier region. The linear fit demonstrates no evidence of aggregation of the protein. **(D)** Normalized Kratky plot calculated using the integrated frames. The shape of the curve is indicative of a flexible particle. **(E)** P(r) function. The calculated Porod volume for the particle is 36 nm^3^. Based on the Porod volume, the molecular mass of the particle is approximately 21 kDa. **(F)** Elongated homology model for AG^(74-173)^. The homology model was based on the SEP3 K domain (4OX0) and secondary structure predictions for AG. Each monomer is depicted as a cartoon and colored blue and green. **(G)** Bent homology model for AG^(74-173)^. Each monomer is depicted as a cartoon and colored blue and green. The homology models **(F,G)** were used for fitting the data using CRYSOL as shown in **(B)**.

The AG^(74-173)^ construct, spanning the complete AG K domain, was designed based on both secondary structure predictions using PSIPRED (Jones, 1999) and homology modeling with the SEP3 structure using SWISS-MODEL. This protein was used in small angle X-ray scattering (SAXS) studies to determine oligomerisation state and conformational flexibility of the AG K domain in solution. The AG^(74-173)^ construct was expressed in *E. coli*, purified from inclusion bodies under denaturing conditions and subsequently refolded. Protein monodispersity and purity were assayed by size exclusion column chromatography (SEC) and SDS-PAGE prior to SAXS experiments. In order to avoid any bias due to protein aggregation or the presence of multiple oligomeric species, the AG^(74-173)^ construct was purified on-line and the complete elution profile measured directly in the X-ray beam (**Figures 7A–C**). The stable radius of gyration (R_g_) across the eluted protein peak corresponding to the highest protein concentration demonstrates that there is one species in solution as the particle size is constant (**Figure 7A**). In contrast to the SEP3 K-domain, which is predominantly tetrameric in solution (Puranik et al., 2014), the AG K-domain is dimeric. Volume calculations based on the histogram of interatomic distances for the particle give a volume of 36 nm^3^, corresponding to a molecular mass of approximately 21 kDa, the molecular mass of an AG^(74-173)^ dimer (**Figure 7E**). AG^(74-173)^ exhibits a great deal of flexibility based on the Kratky plot (**Figure 7D**), which is characteristic of a highly flexible and/or partially disordered protein in solution. In order to further investigate the possible conformations of the AG^(74-173)^ dimer, homology models based on the structure of SEP3 (4OX0) were generated in an elongated and bent conformation (**Figures 7F,G**). CRYSOL fits (**Figure 7B**) were relatively consistent with either particle shape giving chi-squared values of 5.6 and 2.0 for the elongated and bent conformations, respectively. In contrast, the tetrameric SEP3 structure is inconsistent with the recorded data, giving a chi-squared of 38.4 (**Figure 7B**). The R_g_ for both dimeric homology models (3.1 nm for the bent and 3.6 nm for the elongated model) was slightly bigger than the calculated R_g_ of 2.7 nm for the measured data. This variation is attributable to disorder, multiple unmodeled conformations and/or partial unfolding at the termini of the protein. Contamination by a tetramer or soluble aggregates is considered highly unlikely as these species would elute prior to the measured peak and there is no evidence for this in the UV trace or X-ray scattering of the sample.

While possessing glycine residues in the kink region between helices 1 and 2, AG lacks the GlyPro motif seen in SEP1, SEP2 and SEP3. Although it is well-established that AG can form tetrameric complexes, these complexes usually contain a SEP partner. Indeed almost characterized floral organ tetrameric complexes of homeotic MADS TFs from angiosperms to date rely on at least one SEP protein for tetramer formation (Honma and Goto, 2001; Theissen and Saedler, 2001). Thus, the SEPs are able to act as hubs of tetramer formation for other MADS TFs. Because the GlyPro motif forces open helix 2 exposing hydrophobic surfaces, we postulate that the SEP proteins are able to preferentially form tetramers with themselves or other MADS TF proteins and this exposed hydrophobic surface on helix 2 acts as an entropic driving force for oligomerisation.

Some gymnosperm B and C-class MADS TFs are postulated to form tetramers when bound to DNA. *In vitro* studies of GGM2 (*G. gnemon* B-like) and GGM3 (*G. gnemon* C-like) demonstrate that GGM2 can form heterotetramers with GGM3 and that GGM3 is additionally able to homotetramerise when bound to DNA (Wang et al., 2010). Examination of the kink region between helices 1 and 2 as determined from secondary structure predictions and sequence alignments for GGM2 and GGM3 reveals the presence of two glycine residues for GGM2 but no proline. GGM3 has scattered glycines in both the kink region and in the N-terminal portion of helix 2 (**Figure 5A**). We speculate that these glycines will destabilize helix 2 and increase the conformational space the protein is able to sample. Indeed, GGM3 was shown to homotetramerise on DNA with non-optimally spaced binding sites, suggesting additional flexibility of the protein and the tetramerisation interface (Wang et al., 2010). It is likely that the combination of helix destabilization in GGM3 and the relatively plastic kink region in GGM2 is sufficient to allow the formation of tetrameric complexes when the local concentration of the proteins is relatively high as would be the case when bound to adjacent regions of DNA. Further experiments to probe these interactions and extensive mutagenesis studies would be required to fully determine the rules governing tetramerisation. Nascent tetramerisation capabilities are present in at least some species of gymnosperm MADS TFs, though whether tetramerisation is required for proper gene regulation is less clear. However, in angiosperms, interactions mediated by the SEP class of MADS TFs is required for male and female organ specification and reproductive development. The gene duplication event giving rise to the SEPALLATA class of MADS TFs and their central role in organizing the homeotic MADS TFs into functional tetrameric complexes we hypothesize to be a key component in flower origins and evolution.

Taken together, these data suggest that the interaction surfaces and oligomerisation of the MADS TFs is both variable and highly sensitive to even small alterations in amino acid sequence which would allow for the fast evolution of different interactions within the family. By retaining the core DBD, the essential function of the MADS TFs- DNA binding to specific cognate sequences- would be preserved, but mutations in the auxiliary I, K, and C domains would allow for functional plasticity by changing the identity or altering the affinity of protein interaction partners. This model is very similar to what is observed for aLFY, gLFY, and NLY in which the C-terminal DBD is conserved and the auxiliary N-terminal region involved in protein–protein interactions is allowed to vary, likely changing ternary complex formation and tuning downstream gene regulation.

## Conclusion

Small changes in TFs that do not directly affect the DBD can trigger very striking evolutionary developmental changes in an organism. LFY and the MADS TFs illustrate how small changes at the genetic level lead to dramatic alterations and novel functions at the protein level. While the evolutionary origins of the bisexual angiosperm flower are still unclear, major genetic changes - the loss of NLY and the duplication event resulting in the SEPALLATA genes in angiosperms- likely play key roles. How these genetic changes were able to result in morphological changes requires an integrated study incorporating detailed examination of protein structure and biochemistry. By exploring the protein structure-function relationship, particularly for TFs whose activity impacts entire downstream networks, we can begin to understand the molecular basis for evolution. Structural biology offers an important perspective in probing this relationship for the master regulators, LFY and the MADS TFs, and provides a foundation for understanding how alterations in protein structure lead to the evolution of new functions and new morphologies at the organismal level.

## Conflict of Interest Statement

The authors declare that the research was conducted in the absence of any commercial or financial relationships that could be construed as a potential conflict of interest.The Guest Associate Editor Rainer Melzer declares that, despite having collaborated with the author Chloe Zubieta, the review process was handled objectively.
